# Global transcriptomic study of circRNAs expression profile in sorafenib resistant hepatocellular carcinoma cells

**DOI:** 10.7150/jca.39854

**Published:** 2020-03-04

**Authors:** Man-ya Wu, Yan-ping Tang, Jun-jie Liu, Rong Liang, Xiao-ling Luo

**Affiliations:** 1 Research department, Guangxi Medical University Cancer Hospital, Nanning, China; 2 Guangxi Medical University, Nanning, China; 3 Department of Ultrasound, Guangxi Medical University Cancer Hospital, Nanning, China; 4 First Department of Chemotherapy, Guangxi Medical University Cancer Hospital, Nanning, China

**Keywords:** hepatocellular carcinoma, global transcriptomic, sorafenib, circular RNA

## Abstract

The anti-angiogenic drugs represented by sorafenib over the years have always been the first-line treatment of hepatocellular carcinoma (HCC), but the drug resistance has always been a "bottleneck" in curative effect. Recently, aberrant expression of circular RNA (circRNA) is considered to play a crucial role in many types of cancers. However, the genome-wide expression pattern of circRNAs in sorafenib-resistant HCC cells remains unknown. Herein, we identified 1717 differentially expressed circRNAs with 559 up-regulated and 1158 down-regulated (fold change > 2, *P* < 0.05) in sorafenib-resistant (HUH7-S) HCC cells along with 582 differentially expressed circRNAs with 272 up-regulated and 310 down-regulated (fold change > 2, *P* < 0.05) in sorafenib-resistant (HepG2-S) HCC cells, compared to parental sorafenib-sensitive (HUH7, HepG2) HCC cells by high-throughput sequencing. In addition, GO (Gene Ontology) term enrichment analysis results revealed an enrichment for binding and catalytic activity and for biological regulation of metabolic processes in both the Huh7-S and HepG2-S cell lines compared to parental cell lines. Moreover, KEGG (Kyoto Encyclopedia of Genes and Genomes) Pathway analysis of the differentially expressed genes were significantly related to pathways in cancer. Among them, hsa_circ_0006294 and hsa_circ_0035944 expression were consistently down-regulated in resistant HCC cells. Taken together, our data demonstrate, using a global transcriptomic network, that the circRNA expression profile is significantly altered in sorafenib-resistant HCC cells and that the differentially expressed circRNAs may play important functions in HCC sorafenib resistance and HCC progression.

## Introduction

Hepatocellular carcinoma (HCC) is one of the most fatal cancers in the world, ranking fourth globally for death rate 1, and from 2005 to 2015, was the second leading cause of premature death from cancer 2. The main risk factors for HCC include in aflatoxins, hepatitis B virus (HBV), hepatitis C virus (HCV), obesity, excessive alcohol consumption, nonalcoholic fatty liver disease, and smoking, all of which contribute to the development of chronic hepatitis, eventually leading to fibrosis and cirrhosis, held as pre-neoplastic situations 3-5. Surgical resection is still the preferred method for the treatment of liver cancer. However, most patients are at an advanced stage of disease at the time of diagnosis and thus are unsuitable for resection. There are limited therapeutic approaches for advanced liver cancer patients, among which sorafenib, an inhibitor of multiple kinases, is the only molecular targeted drug approved by Food and Drug Administration (FDA) of the United States and European Medicines Evaluation Agency (EMEA) of Europe, for the treatment of patients with advanced liver cancer for whom resection is not an option. Although both the Sorafenib Hepatocellular Carcinoma Assessment Randomized Protocol (SHARP) trial and the Asia-Pacific (AP) trial demonstrated sorafenib-treated patients have an increased median survival time 6, 7, the development of drug resistance hinders progression-free survival.

Circular RNA (circRNA), discovered more than 20 years ago, was originally misread as factors or a byproduct of splicing due to its low expression and sporadic identification in individual genes. However, in recent years, thousands of circRNAs molecules have been discovered *in vivo* due to the development of circRNA purification methods, combined with high-throughput sequencing technologies. According to the sequence composition, circRNAs can be divided into exonic circRNAs, intronic circRNAs, ciRNAs and exonic-intronic circRNAs. Known circRNAs can not only act as competing endogenous RNA (ceRNA) and transcriptional regulators but bind to proteins, such as functioning as a microRNA (miRNA) sponges, combining with RNA binding proteins (RBPs), working as a transcription factor and translation of proteins 8. CircRNAs play important roles in many diseases, including nervous system disorders, atherosclerosis, diabetes and cancer 9. Several studies have found that multiple circRNAs act as oncogenes or tumor suppressors in various cancers. As a tumour suppressor in HCC, circMTO1 regulates P21 expression by targeting miR-9 while circSMARCA5 affects the expression of TIMP3 by sponging miR-181b-5p and miR-17-3p 10, 11. Further, circZKSCAN1 inhibits the growth, migration, and invasion of HCC in cooperation with ZKSCAN1 mRNA 12. Rather, the researchers also explored upstream of circPRKCI promotes the proliferation, migration and invasion of HCC via binding to miR-1324 so that activates the FZD5/Wnt/β-catenin signaling pathway 13. By directly sponging miR-124, circHIPK3 upregulates aquaporin 3 (AQP3) expression and enhances HCC proliferation and migration 14.

Considering these findings, the expression pattern and underlying functions of circRNAs in HCC diagnosis, prognosis and treatment remain to be clarified. Although the role of circRNAs in the onset of the disease has received attention, research into the relationship between circRNAs and chemo-resistance, particularly in sorafenib-resistant HCC, is rare. Herein, we analyzed the differential expression profiles of circRNAs in sorafenib-resistant HCC cells to explore the relationship between the circRNA expression and sorafenib-resistance to provide a preliminary and theoretical basis for the identification of biomarkers for the early diagnosis and malignant progression of HCC.

## Materials and methods

### Cell culture

All cells were obtained from the Institute of Biochemistry and Cell Biology of Chinese Academy of Science (Shanghai, China). Huh-7 cultures were maintained in RPMI-1640 while HepG2 was cultured with Eagle's Minimum Essential Medium supplemented with 10 % fetal bovine serum (FBS) (Gibco, USA) at 37°C in a humidified incubator containing 5 % CO_2_.

### Generation of drug-resistant cells

Cells were treated with 1.5 μM sorafenib (Selleck) after plating into a 6 cm cell culture dish (1×10^5^ cells per dish) for 24 hours. When viable cells remaining attached to the dish, cells respectively treated in various concentrations (1.5, 3.0, 4.5, 6.0, 7.5 and 9.0 μM) were all maintained for 15-21 days. To the end of the fifth month, the cells were becoming stable resistant to sorafenib and re-named Huh-7-S and HepG2-S cells. All experiments were performed in triplicate.

### Cell viability assay

Parental Huh7 and HepG2 and Huh7-S and HepG2-S cells were seeded into a 96-well plate at a density of 1×10^4^ cells/well and treated with sorafenib at concentrations ranging from 0 to 27 μM. After 72 h, viable cells were quantified using the Cell Counting Kit-8 (Dojindo Chemical, Kumamoto, Japan) according to the manufacturer's protocol. Absorbance was measured at 450 nm with a reference wavelength of 600 nm using a microplate reader (Corning, USA). The IC50 values of sorafenib pretreatment on cell viability were examined by the CCK8 assay. All experiments were performed in triplicate.

### RNA isolation and qualification

Total RNA was isolated with TRIzolTM reagent (Invitrogen, USA)/RNeasy Mini Kit (Qiagen) according to the manufacturer's instruction. RNA concentration was measured using Qubit® RNA Assay Kit in Qubit® 2.0 Fluorometer (Life Technologies, CA, USA). RNA purity was assessed using the NanoPhotometer® spectrophotometer (IMPLEN, CA, USA). RNA integrity was checked using the RNA Nano 6000 Assay Kit on the Agilent 2100 Bioanalyzer (Agilent Technologies, Palo Alto, CA, USA), NanoDrop (Thermo Fisher Scientific Inc.) and 1 % agarose gel. Only samples (1μg total RNA) with an RNA integrity number (RIN) of ≥ 7 were subjected to deep sequencing. Next-generation sequencing library preparations were constructed according to the manufacturer's protocol (NEBNext® Ultra™ Directional RNA Library Prep Kit for Illumina®).

### Strand-specific RNA-seq library preparation & sequencing

We prepared a strand-specific RNA-seq library for each sample. Firstly, ribosomal RNA (rRNA) was removed by Ribo-Zero™ rRNA Removal Kit (Human/Mouse/Rat) /(Yeast) / (Bacteria) (Illumina) from 1 μg total RNA. The ribosomal depleted RNA was then fragmented and reverse transcribed. Then, sequencing libraries were generated using NEBNext® UltraTM Directional RNA Library Prep Kit for Illumina® (NEB, USA) following manufacturer's instructions. Briefly, the first strand cDNA synthesis was performed using ProtoScript II Reverse Transcriptase with random primers and Actinomycin D. The second strand cDNA was synthesized using Second Strand Synthesis Enzyme Mix (include dACG-TP/dUTP). The purified double-stranded cDNA by AxyPrep Mag PCR Clean-up (Axygen) was then treated with End Prep Enzyme Mix to repair both ends and add a dA-tailing in one reaction, followed by a T-A ligation to add adaptors to both ends. Size selection of Adaptor-ligated DNA was then performed using AxyPrep Mag PCR Clean-up (Axygen), and fragments of ~360 bp (with the approximate insert size of 300 bp) were recovered. The dUTP-marked second strand was digested with Uracil-Specific Excision Reagent (USER) enzyme (New England Biolabs). Each sample was then amplified by PCR for 11 cycles using P5 and P7 primers, with both primers carrying sequences which can anneal with flow cell to perform bridge PCR and P7 primer carrying a six-base index allowing for multiplexing. Sequences of adaptors were as follows: P7 adapter, 5'- AGATCGGAAGAGCACACGTCTGAACTCCAGTCAC-3'; P5 adapter, 5'-AGATCGGAAGAGCGTCGTGTAGGGAAAGAGTGT-3'. The PCR products were purified using AxyPrep Mag PCR Clean-up (Axygen). Finally, library quality was assessed on the Agilent Bioanalyzer 2100 system, and quantified by Qubit 2.0 Fluorometer (Invitrogen, Carlsbad, CA, USA). The resulting libraries were sequenced on the Illumina HiSeq X Ten System in a 2 × 150 bp paired-end mode.

### Read alignment and transcript assembly

The original image data of the sequencing result was subjected to image base recognition (Base Calling) using the software Bcl2fastq (v2.17.1.14), with preliminary quality analysis. The original sequencing data (Pass Filter Data) was obtained, and the result was stored in the FASTQ file format. The quality of the sequencing data was analyzed using the software FastQC (v0.10.1). The second-generation sequencing data quality statistical software Cutadapt (version 1.9.1) was used to remove the linker and low-quality sequence from the raw data (Pass Filter Data) to obtain clean data for subsequent information analysis. The clean data was compared to the reference genome (GRCH 37) sequence using BWA (version 0.7.12) alignment software, and the results were compared.

### CircRNA identification

The most critical principle for predicting circRNA by high-throughput sequencing is to investigate the sequences of reverse splicing, i.e., back-splicing reads. In the present study, CIRI (V2.0) software was used to predict the position information before and after the formation of circRNA based on the sample sam file for each sample. The CIRI software analyzes the CIGAR values in the SAM format and scans the PCC signals (paired chiastic clipping signals) from the sam file. CircBase is a database built by collecting and integrating published circRNA data. Comparing the predicted circRNA with the positional information of the known circRNA of the circBase database, the newly predicted novel circRNA and the known circRNA can be distinguished. Subsequently, the reads were normalized using SRPBM (spliced reads per billion mapping). Finally, the standardized data is submitted to the GEO database under the number GSE140202.

### Differential expression analysis

Differential expression analysis was performed using the DESeq (V1.6.3) and EdgeR (V3.4.6) Bioconductor package. Data were adjusted using the Benjamini and Hochberg approach for controlling the false discovery rate. The p-value was set to *P* < 0.05 to detect differentially expressed genes.

### Gene Ontology (GO) and pathway analysis

GO-TermFinder (V0.86) was used identifying Gene Ontology (GO) terms that annotate a list of enriched genes with a significant P-value less than 0.05. The ontology has covered domains of biological processes, cellular components and molecular functions. KEGG (Kyoto Encyclopedia of Genes and Genomes) pathway analysis was used to harvest pathway clusters on the molecular interaction and reaction networks in differentially regulated gene profiling. In the present study, significant pathways were identified as those with P‑values < 0.05 and FDR <0.05. The lower values indicated greater significance.

### Quantitative RT-PCR

Real-time PCR was performed using SYBR Premix Ex Taq (TaKaRa) on a LightCycler480 instrument (Roche Diagnostics) according to the manufacturer's protocol. U6 small nuclear RNA was used as an internal normalized reference, and fold changes were calculated by relative quantification (2^-ΔΔCt^). The primers used were as follows: hsa_circ_0006294 forward, 5'-AATCAATGGGAAATGTGAATGTAAC-3'; reverse, 5'-CTGACATCTGAAGGCTCCTCTAATT-3'; hsa_circ_0035944 forward, 5'-CACCATGTCCATTCATAGTAGGGA-3'; reverse, 5'-GAACAGATTCCGGTAGTGAGAATG-3'; hsa_circ_0084663 forward, 5'-GACGGTATCATAGACCAGACCAAG-3'; reverse, 5'-TATTTCTCTGACTCTTGGGCTCAGT-3'; U6 forward, 5'-CTCGCTTCGGCAGCACA-3'; reverse, 5'-AACGCTTCACGAATTTGCGT-3'. All samples were performed by qPCR in triplicate.

### Statistical analysis

Statistical analysis was performed using GraphPad Prism 6.0. CircRNAs with a fold change of more than 2 and *P* values less than 0.05 were considered statistically significant.

## Results

### Establishment of sorafenib-resistant HCC cells

**Figure 1:**
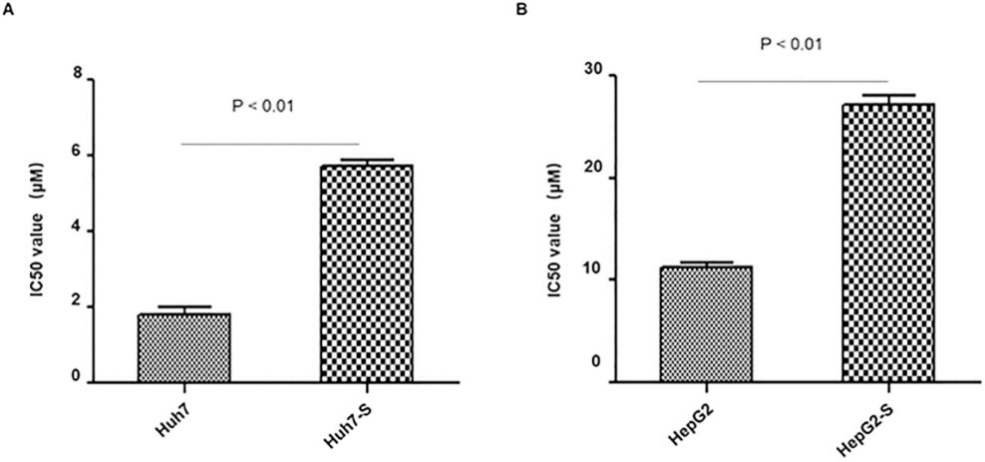
** Resistance of HCC cell lines to sorafenib.** The effects of Huh7 (A) and HepG2 (B) cells and respective resistant cells to sorafenib were assessed by CCK8 assay. The IC50 values for Huh7-S and HepG2-S were 5.71 ± 0.28 μM (3.19-fold) and 27.1 ± 1.72 μM (2.39-fold) respectively, demonstrating significantly higher values than that of parental cells (1.79 ± 0.36 μM and 11.34 ± 0.69 μM) (*p* < 0.01 and *p* < 0.01, respectively). Data are shown as mean ± SEM of three independent experiments.

In order to determine the efficacy of sorafenib resistance in Huh7 and HepG2 cells, IC50 values of parental and sorafenib-resistant HCC cells were determined. IC50 values were significantly higher in sorafenib resistant cell lines, with Huh7-S cells (IC50 = 5.71 ± 0.28 μM) showing a 3.19-fold increased resistance to sorafenib compared to parental Huh7 cells (IC50 = 1.79 ± 0.36 μM; *p*<0.01) (Figure 1A) and HepG2-S cells (IC50 = 27.1 ± 1.72 μM) showing a 2.39-fold increased resistance compared to parental HepG2 cells (IC50 = 11.34 ± 0.69 μM; *p*<0.01) (Figure 1B). Thus, sorafenib-resistant HCC cells were successfully established.

### Screening of differentially expressed circRNAs

**Figure 2:**
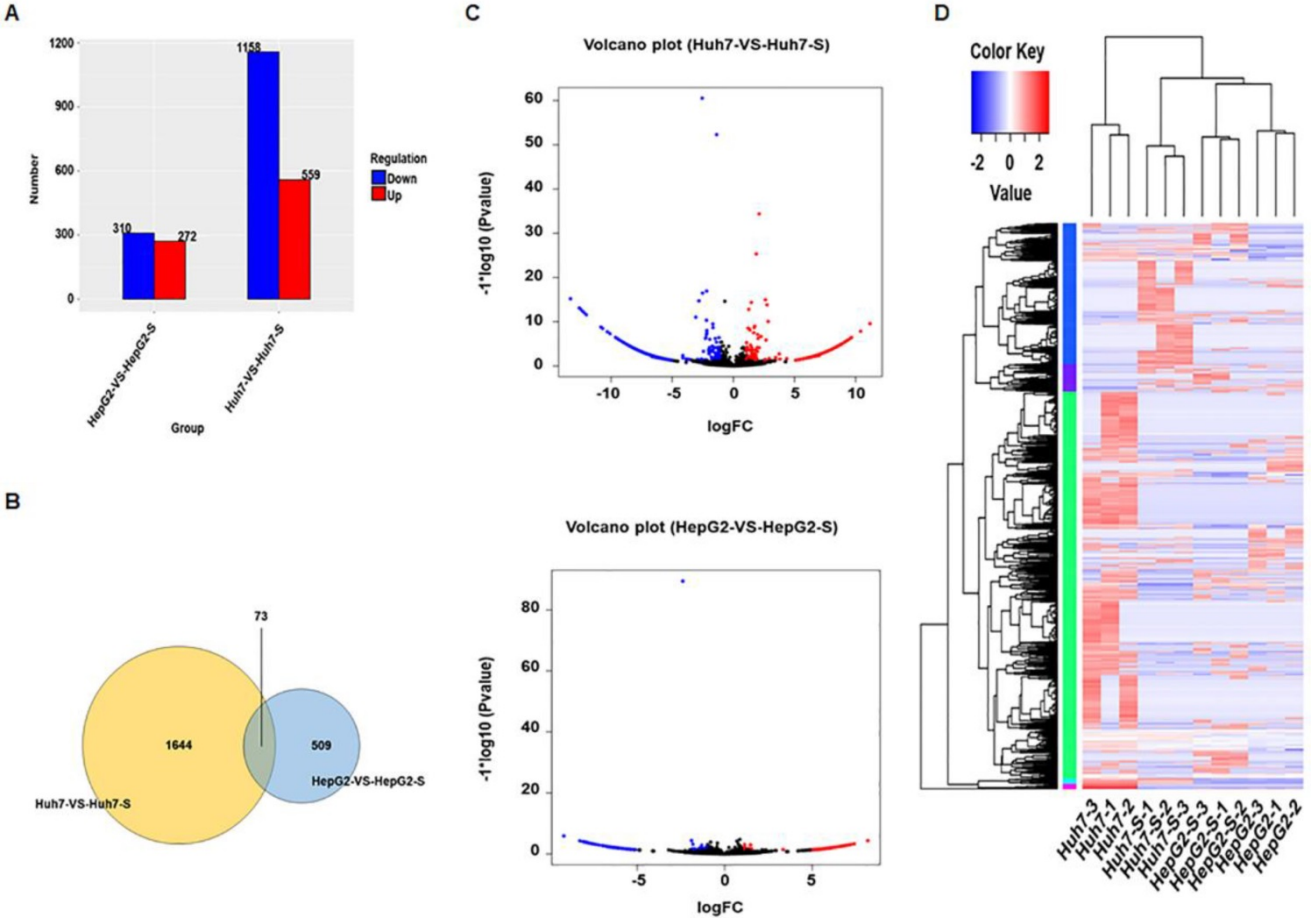
** Screening of differentially expressed circRNAs.** (A) The number of differentially expressed circRNAs in sorafenib-resistant HCC cells compared to the parental cells (blue = down-regulated; red = up-regulated). (B) Venn diagrams of differentially expressed circRNAs in sorafenib-resistant HCC cells compared to the parental cells. (C) Volcano plots of differentially expressed circRNAs between HCC cells and sorafenib-resistant HCC cells. (D) Hierarchical clustering of circRNA expression between HCC cells and sorafenib-resistant HCC cells. The color depth indicated the elevated (Red) and decreased (Blue) expression level across all samples.

Using an Illumina HiSeq instrument according to manufacturer's instructions (Illumina, San Diego, CA, USA), the circRNA expression profile of sorafenib-resistant HCC cells and parental sorafenib-sensitive HCC cells was assessed. In Huh7-S cells, there were 1717 differentially expressed circRNAs compared to the parental Huh7 cells, with 559 up-regulated and 1158 down-regulated circRNAs (Figure 2A). Interestingly, there were only 582 differentially expressed circRNAs in HepG2-S cells compared to the parental HepG2 cells, with 272 up-regulated and 310 down-regulated (Figure 2A). Visualization of the differentially expressed circRNAs using Venn diagrams revealed 73 common circRNAs between the Huh7 and HepG2 comparisons (Figure 2B). The number of significantly altered circRNAs (log fold change < -1 or > 1 and *p* < 0.05) was visualized using a volcano plot (Figure 2C). Hierarchical clustering analysis shows a distinguishable circRNA expression profile among samples (Figure 2D).

### Prediction and functional classification of target genes

**Figure 3:**
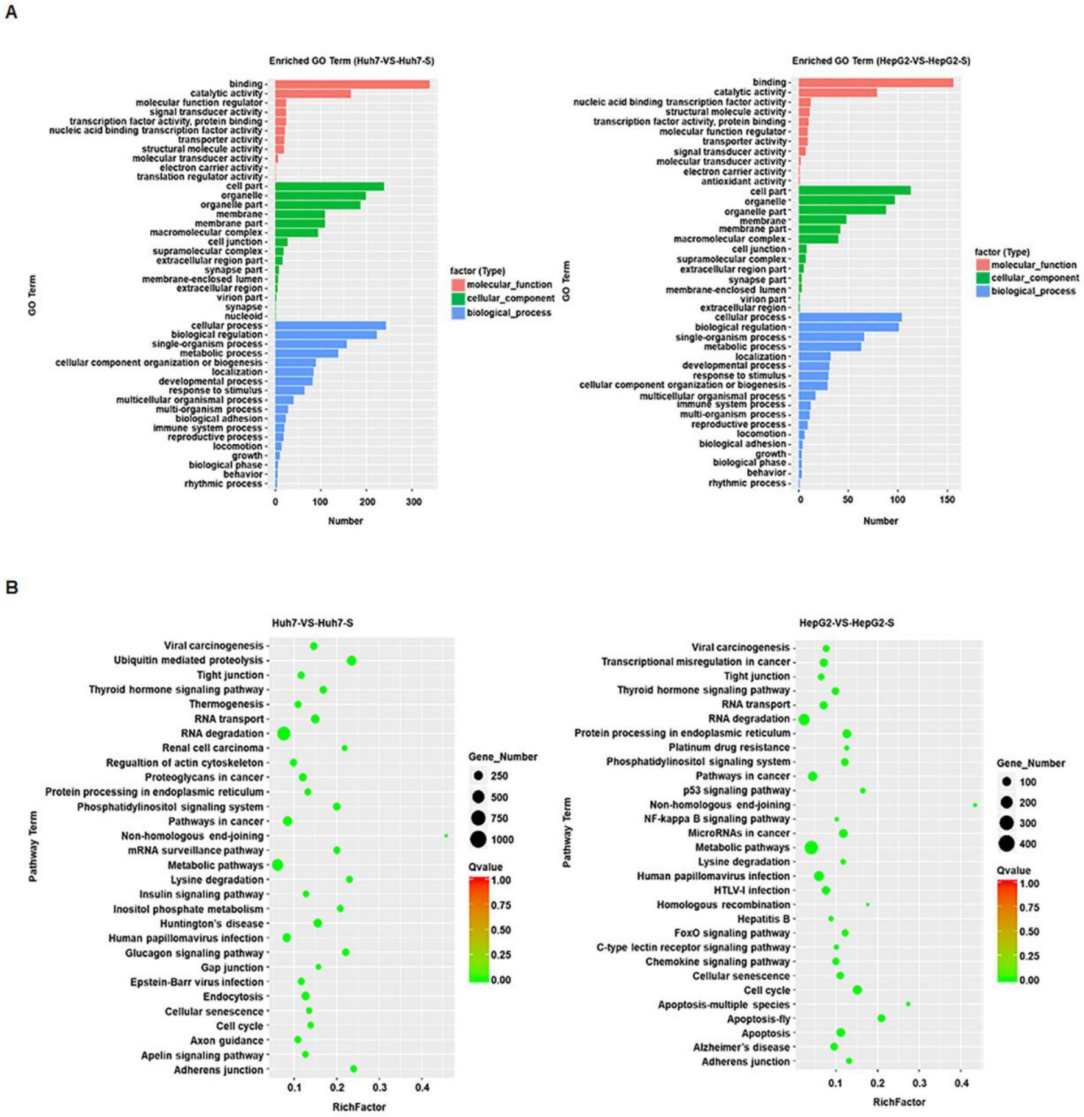
** Prediction and functional classification of target genes**. (A) GO enrichment analysis of significantly differential expressed circRNAs with fold change>2. (B) KEGG pathway analysis of significantly differential expressed circRNAs.

GO term enrichment was performed on the significantly differentially expressed circRNAs in sorafenib-resistant HCC cell lines, compared to the parental cell lines, revealing an enrichment for binding and catalytic activity and for biological regulation of metabolic processes in both the Huh7-S and HepG2-S cell lines compared to parental cell lines (Figure 3A). KEGG molecular functions identified a large proportion of circRNAs involved in RNA degradation common to both cell line comparisons, with non-homologous end joining being the most highly enriched function common to both (Figure 3B).

### Validation of differentially expressed circRNAs

**Figure 4:**
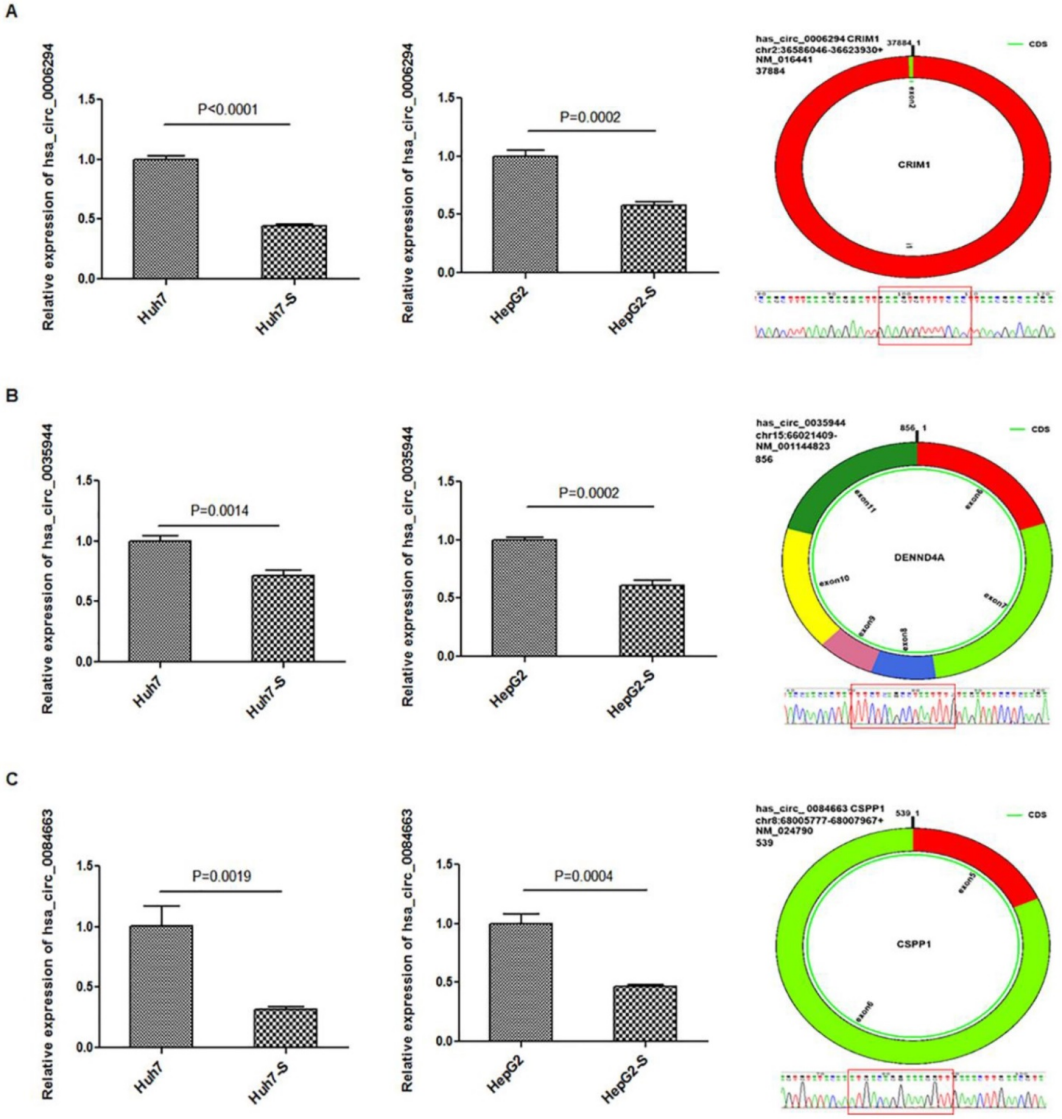
** Validation for circRNAs expression.** The expression levels of A) hsa_circ_0006294; B) hsa_circ_0035944; and C) hsa_circ_0084663 as validated using RT‑qPCR in sorafenib-resistant HCC cells compared to the parental cells. Data are shown as mean ± SEM of three independent experiments.

Three circRNAs, hsa_circ_0006294, hsa_circ_0035944, and hsa_circ_0084663, were down-regulated in both the Huh7-S and HepG2-S cell lines compared to parental control cells and were selected for use in further validation experiments. Using qPCR with U6 small nuclear RNA as the internal standard, the expression levels of hsa_circ_0006294, hsa_circ_0035944 and hsa_circ_0084663 were significantly decreased in both Huh7-S (*p*<0.0001, *p*=0.0014 and *p*=0.0019, respectively) and HepG2-S (*p*=0.0002, *p*=0.0002 and *p*=0.0004, respectively) cell lines compared to the parental controls (Figure 4), indicating that our deep sequencing data are reliable.

## Discussion

Hepatocellular carcinoma (HCC) is one of the most frequent cancers, and to date, there have been very few drugs available that improve survival, however, early detection of HCC provides patients with more favorable outcomes and allows more curative procedures, such as radical primary resection, ablation or liver transplantation to be performed. Sorafenib, one of the most widely used drug for patients with non-resectable tumors, inhibits tumor cell proliferation and angiogenesis and promotes tumor cell apoptosis 15-17, however, the development of its resistance greatly minimizes its therapeutic benefits.

Accumulating evidence indicates that circRNAs may play important roles in regulating cell proliferation, migration, invasion and apoptosis in various cancers. For example, Yanbin, Z* et al.* found that circSAMD4A could promote osteosarcoma proliferation *in vivo* and *in vitro* 18. Circ_0067934 promoted HCC progression via the activation of the Wnt/β-catenin signaling pathway 13 and circRNA_100782 regulated pancreatic carcinoma proliferation through the IL6-STAT3 pathway 19. Further, circZKSCAN1 was revealed to inhibit the growth, migration, and invasion of HCC 12. These findings revealed that circRNAs could be used as potential biomarkers in the diagnosis of cancer or as therapeutic targets by regulating biological processes 20, 21.

Recently, lots of circRNAs have been found to participate in cancer drug resistance and the potential mechanisms of action of the majority of them are being revealed gradually. Zhang et al. found that hsa_circ_001569 was significantly elevated in cisplatin-resistant osteosarcoma cell lines, and this elevation increased cell proliferation and enhanced resistance to cisplatin via activating the Wnt/ β-catenin pathway 22. In prostate cancer, circ_0001427 was found to inhibit enzalutamide resistance by regulating the miR-181c-5p/androgen receptor splicing variant 7 (ARv7) signaling pathway 23. Furthermore, circMTO1 altered cell proliferation, apoptosis, and the resistance of glioblastoma cells to temozolomide by regulating miR-630 24. Though progress has been made into the involvement of circRNAs in multidrug resistant tumors, the expression profile and underlying functions of circRNAs in sorafenib-resistant HCC remain elusive. In present study, we have explored differentially expressed circRNAs in sorafenib-resistant HCC cell lines through high-throughput sequencing and validated three of them. Differentially expressed in sorafenib-resistant HCC cell lines, hsa_circ_0006294, hsa_circ_0035944, and hsa_circ_0084663 were predicted to play an important role in chemoresistance. Since there are no studies about these three circRNAs in human cancer drug resistance, even still unknown whether any circRNA involved in therapy resistance of HCC 25, our findings play a critical role in the chemosensitivity and treatment of HCC.

Besides, among the GO and pathway analysis in this study, the differentially expressed circRNAs were identified as having potential roles in multiple pathways implicated in cancer initiation and progression, including RNA degradation and MDM2-p53 pathways, suggesting that the development of sorafenib resistance in HCC aids tumor progression. Interestingly, the MDM2-p53 pathway has been reported to be of use in the diagnosis and prognosis of hepatocellular carcinoma 26. The expression of MDM2 is elevated in a significant proportion of different cancers, including breast cancer, sarcoma, leukemia, melanoma, and glioblastoma 27. In addition, Zhang* et al.* found that p53 and MDM2 expression levels could be considered as useful indicators for predicting the prognosis of HCC 28. Furthermore, MDM2 knockdown has also been demonstrated to inhibit cancer cell growth and invasion, induce apoptosis, and sensitize HCC cells to sorafenib 29. Unfortunately, the specific targets of circRNAs regulation in sorafenib resistance HCC remain unknown and further *in vitro* and *in vivo* studies are needed to fully reveal the differentially expressed circRNAs and their underlying mechanisms, which may be useful for HCC therapy.

In conclusion, we demonstrated that the circRNA expression profile was significantly altered in sorafenib-resistant HCC cells compared to parental sorafenib-sensitive HCC cells and validated this altered circRNA expression using qRT‑PCR. Additionally, we identified multiple biological processes, cellular components and molecular functions of the differentially expressed circRNAs using GO analysis. Moreover, KEGG pathway analysis revealed that the differentially expressed circRNAs were related to pathways in cancer. Therefore, these findings provide evidence that the circRNA profile of HCC becomes altered during sorafenib resistance and may be of use as potential biomarkers of HCC patients who are developing sorafenib resistance. Further studies into the investigation of the function and mechanism of these differentially expressed circRNAs in sorafenib resistance are required to determine how they are involved in HCC tumour progression.
